# Translation and validation of the audiovisual version of the Montreal cognitive assessment in older adults in Brazil

**DOI:** 10.1186/s12877-023-04553-2

**Published:** 2024-01-03

**Authors:** Cíntia Monteiro Carvalho, Karin Reuwsaat de Andrade, Bruno Costa Poltronieri, Yasmin Guedes de Oliveira, Rafaela Guilherme Ferreira, Erica Woodruff, Rogerio Panizzutti

**Affiliations:** 1 Instituto de Psiquiatria, Universidade Federal do Rio de Janeiro, Avenida Venceslau Brás 71, Rio de Janeiro, RJ 22290140 Brazil; 2 Instituto de Ciências Biomédicas, Universidade Federal do Rio de Janeiro, Rio de Janeiro, Brazil; 3 Instituto Federal de Educação, Ciência e Tecnologia do Rio de Janeiro, Rio de Janeiro, Brazil

**Keywords:** Cognition, Measurement, Mild cognitive impairment

## Abstract

**Background:**

The use of a reliable remote cognitive screening test for older adults is crucial for the diagnosis of cognitive impairment. This study aimed to translate and validate the audiovisual Montreal Cognitive Assessment (MoCA)for older adults in Brazil.

**Methods:**

One hundred and fourteen older adults were recruited from the community and demographic, functional, mood, and cognitive data were collected. Participants were classified into two groups: cognitively healthy or mild cognitive impairment (MCI). Statistical analyses were performed in order to assess the validity of the test and the cutoff score.

**Results:**

The psychometric properties of the audiovisual MoCA showed good convergent validity. The audiovisual MoCA was represented as a unifactorial adjusted model, the composite reliability value was acceptable and a cutoff point of ≥23 reached adequate sensitivity and specificity at 0.77 and 0.92, respectively.

**Conclusions:**

The translated audiovisual MoCA is a valid and reliable cognitive screening test that can be administered remotely in older adults in Brazil. The test demonstrated a great ability to discriminate older adults with MCI from cognitively healthy adults. Future studies should focus on validating the audiovisual MoCA using other target population groups in order to expand the use of this remote screening test.

**Supplementary Information:**

The online version contains supplementary material available at 10.1186/s12877-023-04553-2.

## Background

Mild cognitive impairment (MCI), also called mild neurocognitive disorder according to DSM 5 [1], is a transitional state between normal aging and dementia [2]. MCI is characterized by the presence of cognitive complaints and cognitive impairment (memory and/or other domains), and preserved functional abilities [3, 4]. The conversion rate per year of people with MCI to Alzheimer’s disease varies greatly from 12 to 31% depending on the study. This varied data can be explained by the source of subjects as the main factor, where studies with self-selected participants found higher conversion rates to dementia [5, 6].

Differentiating cases of early dementia from MCI is essential, as there are currently studies involving interventions with lecanemab antibodies in cases of cognitive impairment related to Alzheimer’s dementia. With promising results, both for the delay of cognitive symptoms and the reduction of brain amyloid burden [7]. Given this therapeutic possibility that has recently been available, it has become increasingly important to have sensitive diagnostic support through tools to detect MCI. The Montreal Cognitive Assessment (MoCA) is a neuropsychological tool, widely used by professionals in clinical settings [8], created as a screening test to detect MCI. Among cognitive screening tests, the Mini-Mental State Examination (MMSE) and MoCA are considered the gold standards for detecting cognitive impairment. However, MoCA has greater diagnostic accuracy in discriminating between patients with MCI and Alzheimer’s disease [9, 10] and it is recommended as a cognitive screening test for older adults by the Department of Cognitive Neurology and Aging of the Brazilian Academy of Neurology [11]. In Brazil, the MoCA test was originally validated in 2009 and the results showed good psychometric properties [12].

In recent years, telemedicine has proven to be relevant, especially after the COVID-19 pandemic [13] becoming part of routine clinical practice, considerably improving access to healthcare services in an effective and efficient manner [14]. Among the advantages of using videoconferencing tools, is the possibility of screening the cognitive performance of people with mobility restrictions, who live in distant areas or places without access to specialized professionals [15]. Video conferencing involves the synchronized transfer of visual and auditory information, requires an efficient internet connection between the people involved and the software needed to carry out this type of assessment, does not require high costs and is easy to use.

Studies have demonstrated that cognitive screening instruments administered remotely have high psychometric and diagnostic quality, being considered comparable to tests carried out in person [16]. Among the different versions of MoCA tests available [17, 18], there is the audiovisual MoCA version, which allows it to be applied remotely in telemedicine. The use of this version has been studied by some populations and the authors found that it is a valid alternative to face-to-face assessment [19, 20]. However, the number of validation studies of the audiovisual MoCA in older adults is still scarce. Therefore, it is crucial that new research examines the sociodemographic, cultural and linguistic differences in the remote application of these instruments in the Brazilian population, in addition to investigating the psychometric quality [21].

Given the relevance of this instrument and the evidence of its effectiveness in remote application in other countries, we sought to translate and validate the audiovisual MoCA into Brazilian Portuguese, aiming for its use in telemedicine. Therefore, the objective of this study was to translate and validate the audiovisual MoCA, a cognitive screening instrument, that contributes significantly to the differential diagnosis of Brazilian older adults who present some type of cognitive impairment, whether MCI or dementia.

## Methods

The translation and validation of the audiovisual version of the MoCA in older adults in Brazil was carried out between July 2020 and October 2022, following two steps: 1) translation of the test and the establishment of content validity, and 2) the evaluation of test psychometric properties. This study followed the guidelines proposed by Guillemin [22] and the recommendations of the American Psychological Association [23].

### Translation and content validation

This study began after the authorization and an agreement of terms between the author of the test [8] and the authors of the present study. The translation and content validation process consisted of six steps, which are described below:

*Initial translation:* two translations in Brazilian Portuguese were produced independently by two bilingual researchers; one was certified as a sworn translator and was unfamiliar with the material translated and the concepts involved; and the other was a bilingual specialist in neuropsychology who had extensive experience with cognitive assessments.

*Synthesis of the translations:* the two translators discussed and elaborated a synthesis of the two translated versions, and a third researcher resolved any discrepancies.

*Back translation:* the Brazilian Portuguese translation was back-translated independently into English by two bilingual specialists in neuropsychology. The translators compared and discussed the two versions to obtain an agreed back-translated version.

*Committee review:* five experts were asked to evaluate the Brazilian Portuguese MoCA audiovisual version independently for content validity. Most of the experts were female (80%), two were occupational therapists, two were psychologists, and one was a psychiatrist. The experts were invited by email to participate in the study, they were informed of the objectives and methods of the study and also about how to carry out the content validity assessment. After acceptance of participation, an electronic form was sent by email. The experts were asked to record their scores on a 5-point Likert scale, ranging from 1 to 5. They compared the original and back-translated versions. For each item, the experts assessed independently the language clarity, appropriateness, and understanding. The experts were also invited to provide additional comments on each scale item. The content validity coefficient was computed to quantify content validity. A cutoff score of 0.90 for each item was used to indicate a satisfactory level of language clarity of content, appropriateness, and understanding.

*Pre-testing:* the preliminary version of the Brazilian Portuguese audiovisual MoCA was applied to a sample of 30 healthy older adults (aged 60 and over). Most of them were female (86%), and the mean age was 66 (standard deviation [SD], 5.38). The older adults were invited to participate in the study by email or by phone call, wherein they were informed about the objectives and methods of the study. The content validity assessment was performed through video call by a member of the research group, who read each item and asked the older adult to rate on a 5-point Likert scale, ranging from 1 to 5. For each item, the older adults assessed independently the language clarity, appropriateness, and understanding and they were also invited to provide additional comments on each scale item. The content validity coefficient was computed to quantify content validity. The cutoff score of 0.90 for each item was used to indicate a satisfactory level of language clarity of content, appropriateness, and understanding.

*Submission to the authors:* at this stage, the final Brazilian Portuguese and back-translated versions were sent to the authors of the original MoCA, who provided feedback and agreed with the final versions, and then published the test on the official website MoCA - Cognitive Assessment [24].

### Evaluation of psychometric properties of the MoCA

#### Participants

Participants were recruited from the community, through referrals from professionals in the field and posts on social media (Instagram and Facebook), between March 2021 and October 2022. We invited older adults (aged 60 or above) to participate in the study and included those who: 1) provided written informed consent; and 2) had a normal or corrected vision and hearing. Participants were excluded if they: 1) were diagnosed with dementia; 2) were dependent on daily living activities; 3) had major medical disorders that precluded participation in the study; and/or 4) did not have access to the internet or a computer.

All subjects underwent an evaluation consisting of full medical clinical history, and assessments of cognition, functionality, and symptoms of anxiety and depression. Subjects were diagnosed according to DSM 5 [1, 25]. The diagnosis was confirmed by a trained psychiatrist (R.P.)

#### Instruments

Clinical history, cognitive, functionality, and symptoms of anxiety and depression assessments were collected by video call. The audiovisual MoCA was applied to assess six cognitive domains: attention (sustained attention task, subtraction task, digit span); executive functions, (trail-making test B, phonemic verbal fluency, verbal abstraction); visuospatial abilities (cube, clock drawing); language (nomination, sentence repetition, phonemic verbal fluency as mentioned above); memory (delayed recall); and orientation .

The test begins with the short trail-making test, administered orally through screen sharing. In this segment, the examiner presents the short trail-making test and the example, followed by a request for the participant to indicate the arrow’s next position while adhering to ascending and alphabetical orders (1 point). Subsequently, for the bed copy task, the examiner shares a model with the participant via screen sharing, and asks the participant to draw the model, after drawing the participant displays his own drawing on camera for scoring purposes (1 point). This is succeeded by the clock drawing task, during which participants exhibit their clock drawings on camera after drawing it (3 points). Naming is evaluated through the identification of three animal figures presented on the screen via screen sharing. (a horse, a tiger and a duck, totalling 3 points). Following this, attention is assessed with a forward and backward digit span task (2 points), a letter tapping task (1 point) and a Serial 7-subtraction task (3 points). The participant is then instructed to repeat two complex sentences (2 points) and subsequently perform a phonemic fluency task (1 point was awarded if > 11 words were produced in one minute). Abstraction is assessed with a two-item similarity task (2 points), and memory is evaluated by a delayed recall of a five-word list learned previously (5 points). During the two readings of the list, participants is instructed not to write down the words. Lastly, in the orientation subscale, standard administration involves participants informing the assessor of their current location and the date of the test day (6 points for the orientation subscore). The MoCA audiovisual incorporates additional aspects and instructions related to internet connection quality, camera activation, and guidance to avoid checking their watch or a clock, along with instructions for drawing a clock (more information about the differences between both versions of MoCA can be accessed in Supplementary Table 1 and detailed information about the instructions can be found in the website [23].

The score is obtained by adding the points of each successfully completed task, ranging from 0 to 30 points, with higher scores indicating better cognitive performance. An additional point is given to individuals with 12 or fewer years of education to correct for educational effects, as found in the original study [8].

The other neuropsychometric tests applied were: the Semantic Verbal Fluency (SVF) test, which consists of the generation of words in a limited amount of time starting with a semantic category [26]; the Rey Auditory Verbal Learning Test (RAVLT), which involves repeated auditory presentation and recall of a word list [27]; and the MMSE, which enables the quick assessment of spatiotemporal orientation, attention, verbal memory, language, and praxis [9].

To assess mood, we applied the Geriatric Anxiety Inventory (GAI), which consists of 20 agree/disagree items to evaluate anxiety symptoms [28], and the 15-item version of the Geriatric Depression Scale (GDS) to measure the symptoms of depression [29].

To determine functionality, we used the Katz Index of Independence in Activities of Daily Living, which ranks adequacy of performance in the activities of daily living [30].

### Statistical analysis

Descriptive analyses were used for the sample characterization. The sample size of the CH and MCI groups was estimated for achieving acceptable estimates of sensitivity and specificity (minimum of 70%) using likelihood ratio contingency tables, considering a unilateral hypothesis, and prevalence and ratio of false positives, considering Fleiss’s correction. The chi-squared (χ^2^) test and the two-sample t-test were performed to enable comparisons between the two groups. A one-way analysis of covariance (ANCOVA) was performed to control for age and education with the scores of MoCA and MMSE.

Confirmatory factor analysis was conducted to provide further evidence of the factor structure of the audiovisual MoCA. The analysis was implemented using the estimation method of Robust Diagonally Weighted Least Squares (RDWLS) [31, 32]. The additional fit measures used were: χ^2^; χ^2^/df; Comparative Fit Index (CFI); Tucker–Lewis Index (TLI); Standardized Root Mean Square Residual (SRMR); and Root Mean Square Error of Approximation (RMSEA). The χ^2^ values should not be significant; the χ^2^/gl ratio must be ≤5, preferably ≤3; the CFI and TLI values must be ≥0.9, preferably > 0.95; and the RMSEA values must be ≤0.08, preferably ≤0.06, with a confidence interval (upper limit) of ≤0.10 [32].

The convergent validity was determined through Pearson’s correlation analysis between MoCA and MMSE. The association between the audiovisual MoCA score and age, and the association between the audiovisual MoCA score and educational level, were also analysed through Pearson’s correlation, where r < 0.4 values represented weak correlation; 0.4 > r < 0.5, moderate correlation; and r > 0.5, strong correlation [33]. Reliability was assessed through a composite reliability analysis of the factors, where a value > 0.70 is acceptable [34].

The diagnostic accuracy of the audiovisual MoCA for MCI and CH individuals was assessed through the receiver operating characteristic (ROC) curve analysis, and the area under the curve (AUC) was determined.

All statistical analysis was performed using version 28 of the SPSS statistics software (IBM SPSS Statistics for Windows, NY: IBM Corp.) and Jeffreys’s Amazing Statistics Program (JASP) version 0.16.4.

## Results

### Sample characterization

The translated audiovisual MoCA was applied to 114 older adults via teleconference; 79 participants were cognitively healthy and 35 were diagnosed as MCI.

In the total sample, the mean age was 69 (SD = 6.8), the majority of participants were female (81.6%), and the mean number of education years was 15.4 (SD = 3.9). Demographic and clinical information of participants are described in Table 1.

**Table 1:** Demographic and clinical information

Subjects characteristics	Total sample (*n* = 114)	Cognitively Healthy (*n* = 79)	MCI (*n* = 35)	*p*-value
Age, years (SD)	69 (6.8)	67 (5.4)	73.4 (7.7)	< 0.001
Female (%)	81.6	84.8	74.3	0.18
Education, years (SD)	15.4 (3.9)	16.2 (2.2)	13.6 (5.9)	< 0.001
MoCA (SD)	24.9 (3.5)	26.5 (2)	21.5 (3.7)	< 0.001
MMSE (SD)	27.1 (2.4)	28.2 (1.6)	25 (2.5)	< 0.001
GDS (SD)	3.8 (2.4)	3.7 (2.9)	3.9 (2.4)	0.71
GAI (SD)	6 (3.7)	6.3 (3.3)	5.4 (4.4)	0.12
Katz index (SD)	5.9 (0.4)	5.9 (0.2)	5.8 (0.6)	0.18

*MCI* Mild cognitive impairment, *M* Mean, *SD* Standard deviation, *MoCA* Montreal cognitive assessment, *MMSE* Mini mental, *GSD* Geriatric Depression Scale, *GAI* Geriatric Anxiety Inventory, *Katz index* Independence in Activities of Daily Living

There was a significant difference in age, years of education and cognition between healthy controls and the MCI group. The groups did not differ in terms of symptoms of depression and anxiety, as assessed by the GDS and GAI, respectively, and basic activities of daily living assessed by the Katz index. In conclusion, the MCI group was older, had a lower educational level, and worse cognition and functionality in comparison to the cognitively healthy group.

To exclude the confounding factors of age and education, we performed an ANCOVA analysis to control the effects of these variables on the MoCA and MMSE results. The average score of MoCA in the two groups differed significantly from each other (Table 1) and remained significant after controlling for the effects of education (F(1.111) = 64.622, *p* < 0.001, η2 = 0.368) and age (F(1.111) = 49.918, *p* < 0.001, η2 0.310). Similarly, the average score of MMSE in the two groups differed significantly from each other (Table 1) and remained significant after controlling for the effects of education (F(1.88) = 41.610, *p* < 0.001, η2 = 0.321) and age (F(1.88) = 28.989, *p* < 0.001, η2 = 0.248).

### Psychometric properties

To assess convergent validity, we performed a correlation between the audiovisual MoCA scores and the MMSE scores; we found a high positive correlation between the scores from both tests (*r* = 0.72, *p* < 0.001), indicating good convergent validity. Thus, to determine the factor structure of the audiovisual MoCA we carried out confirmatory factor analysis. Based on the original conceptual model proposed by the authors of the MoCA [8], which aims to screen global cognition, and in previous studies carried out by Duro [35] and Mai Do [36], we performed a one-factor model composed of six cognitive domains (memory, visuospatial abilities, executive functions, language, orientation, and attention) which constituted a global factor “Global Cognition”. Table 2 demonstrates the confirmatory factor analysis results.

**Table 2:** MoCA confirmatory factor analysis results

	*χ*2(df)	*p*	*χ*2*/df*	CFI	TLI	RMSEA (90% CI)
Original model	21.037 (9)	0.012	2.337	0.964	0.941	0.109 (0.048–0.170)
**Adjusted model**	**12.007 (8)**	**0.015**	**1.500**	**0.988**	**0.978**	**0.067 (0.000–0.139)**

*χ2* Chi-square test statistic, *df *Degrees of freedom, *χ2/df *Relative Chi-square, *CFI *Comparative Fit Index, *SRMR *Standardized Root Mean Residual, *TLI *Tucker-Lewis Index, *RMSEA *Root mean square error of approximation

**Fig. 1:**
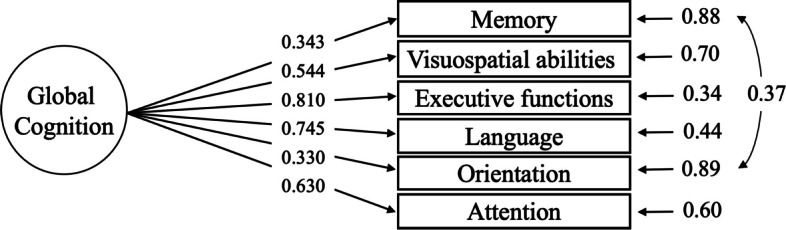
Confirmatory factor analysis model demonstrating the factor structure of the test

The unidimensional structure showed contradictory fit results, as this model had significant chi-square values and an RMSEA value of > 0.08. To better inspect the results, we evaluated the modification indices (MI), which showed high residual covariance between memory and orientation items (MI = 8.970). Figure 1 shows the structure, factor loadings, and residual variance of items.

By inserting this residual covariance in the model, there was an improvement in all fit indices (Table 2), as the RMSEA became acceptable (≤0.08), indicating excellent factorial adequacy. Subsequently, we performed a reliability analysis using the composite reliability of the factors, and the results were acceptable (0.76).

### Cutoff points

**Fig. 2:**
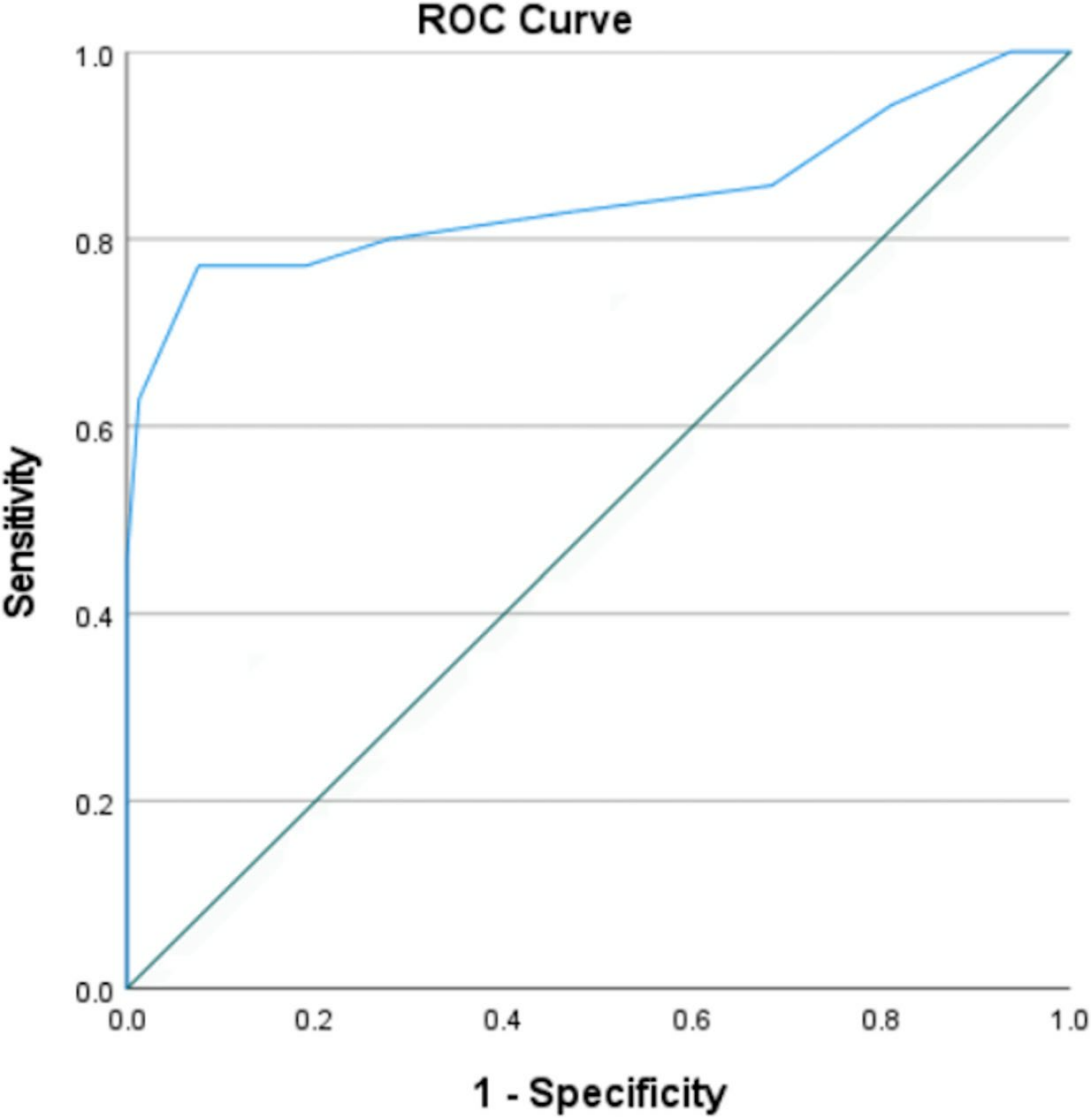
Representation of the ROC curve of the audiovisual MoCA in discriminating the older adults with mild cognitive impairment from the cognitively healthy older adults

To assess the sensitivity and specificity of the audiovisual MoCA in discriminating the older adults with MCI from the CH adults, we performed ROC curve analysis. This resulted in a statistically significant curve, demonstrating that, when chosen randomly, 84.5% (CI 0.747–0.943) of the clinical cases will present scores lower than non-clinical cases in the audiovisual MoCA (Fig. 2: AUC = 0.845, EP = 0.05; *p* < 0.000; 95% CI = 0.747–0.943).

The cutoff score that maximized sensitivity and specificity was 23/24 (i.e., scores up to 23 and from 24 onwards), with a sensitivity of 0.77 and specificity of 0.92.

Finally, to investigate whether age or education affected the test scores, we performed a correlation analysis between the audiovisual MoCA scores and years of education (*r* = 0.364; *p* < 0.001); and the audiovisual MoCA scores and age (*r* = − 0.43; *p* < 0.001). The results showed a weak positive correlation for education, indicating that the scores were slightly affected by this variable, and a significant moderate, negative correlation for age, showing that the higher the age, the lower the score found in the audiovisual MoCA test.

## Discussion

The objective of this study was to translate and validate the MoCA in a audiovisual format in older adults in Brazil. The final version of the test showed good, consistent psychometric properties and demonstrated a great ability to discriminate older adults with MCI from cognitively healthy older adults. Considering the rapid increase of the older adult population in several regions of the world, including Brazil [37], reliable and structured instruments for cognitive screening that can be administered remotely are needed. Those instruments can contribute to the assessment of individuals with limited mobility or in regions of scarce resources. Through this study, we provide a validated tool for the remote screening of cognition which, in addition to the clinical implications, should generate new and relevant scientific data in the field of aging in Brazil.

Results of audiovisual MoCA and MMSE, showed a high correlation, indicating convergent validity with another test that measures the same construct. Although high correlation coefficients are expected, convergent validity analyszes from other studies that correlated the same tests have found great variability, with outcomes ranging from 0.65 to 0.93 [8, 10, 35, 38–40]. A MoCA validation study conducted in Brazil [12] reported a similar result (r = 0.74) to ours.

Although several MoCA validation studies have been conducted in different languages and population groups, few performed the confirmatory factor analysis to assess the factor structure of the test. Our findings indicate that among older adults in Brazil, the remote audiovisual MoCA is represented as a unifactorial adjusted model. This model was originally proposed by the authors of the MoCA [8] and is composed of a global factor “Global Cognition” that includes six cognitive domains (memory, visuospatial abilities, executive functions, language, orientation, and attention). In our results, after inserting the residual covariance between memory and orientation, all factor loadings were within the acceptable range, demonstrating that this model is suitable. A study conducted by Duro et al. [35] also tested the one-factor model and found a similar result in the validation of the MoCA in Portugal, including adjusting the same correlation between the residual variances of memory and orientation. Another MoCA Vietnamese validation study also tested the same model, and the test performed statistically better with covariances between language and executive functions [36]. Therefore, our results confirm the theoretical structure of the test, which considers MoCA an instrument for global cognitive screening [8].

The composite reliability of the factors was also acceptable, indicating great internal consistency. Different from most MoCA validation studies that used Cronbach’s alpha value, we opted for composite reliability analysis due to the strong limitations of the use of Cronbach’s α [41–43], owing to its restrictive assumptions.

In addition, AUC values in the ROC curve proved to be satisfactory indicating the good accuracy of the audiovisual MoCA for discriminating individuals with MCI from cognitively healthy. These results are in line with a previous meta-analysis of MoCA validation studies that determined the cutoff score for differentiating healthy aging from MCI as 23 [36], as opposed to 26 initially recommended by Nasreddine et al. [8]. However, caution is needed when using this cutoff score, especially in the context of critical clinical decisions, as the MCI and cognitively healthy groups differ significantly in terms of age and education. In this study, we performed an analysis of covariance to control the effects of these variables on the MoCA and MMSE results, and the difference between the groups remained even when controlling for age. A meta-analysis study on the re-examination of the MoCA cut-off score [44] discusses that MoCA performance has been shown to be influenced by age, years of formal education, and cultural background and that it would be wise to consult normative data to improve accuracy in interpretation of the MoCA cut scores.

In our study, the results of the remote audiovisual MoCA were influenced by age. As in other studies [15, 45], older adults had lower scores than younger ones. This result is in accordance with other studies that have shown that the risk of MCI and cognitive decline increases with aging [46]. Age-related changes in brain structure and function can be associated with cognitive decline even in normal aging [47]. Therefore, such changes are expected to affect performance on the MoCA and other cognitive tests.

Finally, the audiovisual MoCA results were weakly affected by education level, as indicated by the authors of the test [8], we added one point to the total score for those individuals with low schooling to minimize the influence of education. Our findings, as well as other studies [40, 48, 49], support those of Nasreddine et al. [8]. A previous study on normative data for the audiovisual MoCA evaluated the influence of sociodemographic characteristics on performance in the test, finding robust correlations between education and age. Not only older individuals, but those with less education performed worse on the MoCA [8]. In addition to MoCA performance, education has large effects on cognition in older age, even when controlling for genetic factors [50].

Some limitations of this study must be addressed. The clinical diagnosis of MCI was made based on the anamnesis, information about clinical conditions and the remote application of neuropsychological tests. Although there are studies in the literature that address the use of neuropsychological tests remotely, such as the study that determines the reliability of the Mini-Mental State Examination (MMSE) administration via telehealth [51] and another study that aimed to collect evidence of the validity of a remote cognitive battery that uses the same neuropsychological tests used in our study [52], to the best of our knowledge this is the first validation study of a remote cognitive test in Brazil, therefore it was not possible to apply validated tests, on the other hand, this point further highlights the relevance and innovation of this study.

Despite the fact that we tried to diversify sociodemographic characteristics, our sample is mostly composed of participants with a high level of education and access to technological resources, which can be attributed to the characteristics of recruitment. Therefore, the generalization of these results to other target populations should be done cautiously.

Another limitation is that it was not possible to carry out an in-person re-test with the validated paper-based MoCA version in order to compare the results intra-subject. However, we must take into account that at the moment of the assessments we were in a critical period of the pandemic of COVID and social isolation with no prospect of returning to in-person services. With this, our investigation was the first online validation study aiming to enable a standardized application of cognitive screening tests.

## Conclusions

The audiovisual MoCA is a valid and reliable cognitive screening test that can be administered in older adults through videoconference, which makes it possible to apply it even over great distances. Furthermore, this is an inexpensive and easy-to-use tool. The cutoff point of ≥23 reached an adequate sensitivity of 0.77 and specificity of 0.92. The findings of this study have a relevant impact both for scientific research and for the clinical area, since the MoCA test is the most widely used test for cognitive screening in older adults. Future studies should focus on validating the audiovisual MoCA to other target population groups, as well as validating other tests commonly administered in older adults as remote screening tools to expand the repertoire of these tools and strengthen the growth of telemedicine.

### Supplementary Information

**Additional file 1: Table S1.** Detailed differences between MoCA Full, face-to-face and Audiovisual MoCA.

## Data Availability

The datasets used and/or analyzed during the current study are available from the corresponding author (RP) on reasonable request.

## Abbreviations

AUC: Area Under the Curve
CFI: Comparative Fit Index
CH: Cognitively Healthy
DSM: Diagnostic and Statistical Manual of Mental Disorders
GAI: Geriatric Anxiety Inventory
GDS: Geriatric Depression Scale
MoCA: Montreal Cognitive Assessment
MCI: Mild Cognitive Impairment
MI: Modification Indices
MMSE: Mini-Mental State Examination
RAVLT: Rey Auditory Verbal Learning Test
RDWLS: Robust Diagonally Weighted Least Squares
RMSEA: Root Mean Square Error of Approximation
ROC: Receiver Operating Characteristic
SD: Standard Deviation
SRMR: Standardized Root Mean Square Residual
SVF: Semantic Verbal Fluency
