# Supplementary material for: Translation and validation of the audiovisual version of the Montreal cognitive assessment in older adults in Brazil
Source: BMC Geriatr. 2024 Jan 3;24:10. doi: 10.1186/s12877-023-04553-2 (PMC10765722; doi:10.1186/s12877-023-04553-2)
Supplement: Supplementary file 1 — Additional file 1: Table S1. Detailed differences between MoCA Full, face-to-face and Audiovisual MoCA. [file 12877_2023_4553_MOESM1_ESM.docx]

Supplementary Table 1: Detailed differences between MoCA Full, face-to-face and Audiovisual MoCA.

| Task | MoCA full, face to face | Audiovisual MoCA |
| --- | --- | --- |
| Alternating Trail Making | Administered with paper and pen. The participant needs to draw a line between numbers and letters. | Administered orally through screen sharing. The participant speaks the correct order between numbers and letters. |
| Visuoconstructional Skills (cube/bed) | The examiner shows a picture of a cube. The participant should draw a cube and then give it to the examiner. | The examiner screen sharing a picture of a bed. The participant should draw a bed and then show in camera to the examinator the picture. |
| Visuoconstructional Skills (Draw clock) | The participant should draw a clock showing 10h05 and then show it to the examiner. | The participant should draw a clock showing 11h10 and then show the examiner in the camera the picture. |
| Naming | The examiner shows a picture with three animals and asks the participant to say, one by one, the names of the animals printed on the paper: lion, rhinoceros, camel. | The examiner screen shares a picture with three animals and asks the participant to say, one by one, the names of the animals on the screen: horse, lion, duck. |
| Forward Digit Span | The examiner speaks some numbers: 2-1-8-5-4, and the participant must say the digits in direct order. | The examiner speaks some numbers: 2-1-8-5-4, and the participant must say digits in direct order. |
| Backward Digit Span | The examiner speaks some numbers: 7-4-2, and the participant must say digits in indirect order. | The examiner speaks some numbers: 7-4-2, and the participant must say digits in indirect order. |
| Vigilance | Every time the participant hears the letter "A", he must hit the table with his hand. | Every time the participant hears the letter "A", they must clap their hands facing the camera. |
| Serial 7 subtraction | The participant is asked to subtract 7 from 100 and continue subtracting 7 from the answer until the examiner stops. | The participant is asked to subtract 7 from 60 and continue subtracting 7 from the answer until the examiner stops. |
| Sentence repetition | The participant is asked to repeat the phrases spoken by the examiner. The sentences in Portuguese are:  "Eu somente sei que é João quem será ajudado hoje” (I only know that it is João who will be helped today).  “O gato sempre se esconde embaixo do sofá quando o cachorro está na sala” (The cat always hides under the sofa when the dog is in the living room). | The participant is asked to repeat the phrases spoken by the examiner. The sentences in Portuguese are:  "A criança passeou com seu cachorro no parque depois da meia-noite” (The child walked his dog in the park after midnight).  "O artista terminou sua pintura no momento certo para a exposição.” (The artist finished his painting at the right time for the exhibition). |
| Verbal fluency | The participant must speak as many words starting with the letter "F" as possible, except names of people, places, numbers and different verb conjugations. | The participant must speak as many words starting with the letter "B" as possible, except names of people, places, numbers and different verb conjugations. |
| Abstraction | The participant is asked to say the category that the words "train and bicycle" and "clock and ruler" belong to. | The participant is asked to name the category that the words "Hammer and screwdriver" and "match and light bulb" belong to. |
| Delayed Recall | The participant is asked to recall the words said at the beginning of the test. The requested words are: "rosto" (face), "veludo" (velvet), "igreja" (church), "margarida" (daisy), and "vermelho" (red). | The participant is asked to recall the words said at the beginning of the test. The requested words are: "perna" (leg), "algodão" (cotton), "escola" (school), "tomate" (tomate) and "branco" (white). |
| Orientation | The participant is asked to answer the date on which the assessment is taking place, the day of the week, month, year, place where the participant is located and city. | The participant is asked to close their eyes and answer the date on which the assessment is taking place, the day of the week, month, year, which institution the examiner belongs to or works at and which city this institution is located in. The information about the city where the institution is located is mentioned before the test starts. |

Note: (translation of words or phrases into English).
